# A Spectral Fiedler Field-based Contrast Platform for Imaging of Nanoparticles in Colon Tumor

**DOI:** 10.1038/s41598-018-29675-1

**Published:** 2018-07-30

**Authors:** Chenang Liu, Ankur Kapoor, Joshua VanOsdol, Kalyani Ektate, Zhenyu Kong, Ashish Ranjan

**Affiliations:** 1 Grado Department of Industrial and Systems Engineering, Virginia Tech, Blacksburg, Virginia USA; 2 Siemens Healthineers, Princeton, New Jersey USA; 3 Center for Veterinary Health Sciences, Oklahoma State University, Stillwater, Oklahoma USA

## Abstract

The temporal and spatial patterns of nanoparticle that ferry both imaging and therapeutic agent in solid tumors is significantly influenced by target tissue movement, low spatial resolution, and inability to accurately define regions of interest (ROI) at certain tissue depths. These combine to limit and define nanoparticle untreated regions in tumors. Utilizing graph and matrix theories, the objective of this project was to develop a novel spectral Fiedler field (SFF) based-computational technology for nanoparticle mapping in tumors. The novelty of SFF lies in the utilization of the changes in the tumor topology from baseline for contrast variation assessment. Data suggest that SFF can enhance the spatiotemporal contrast compared to conventional method by 2–3 folds in tumors. Additionally, the SFF contrast is readily translatable for assessment of tumor drug distribution. Thus, our SFF computational platform has the potential for integration into devices that allow contrast and drug delivery applications.

## Introduction

In efforts to improve solid tumor imaging, and enable image-guided drug delivery (IGDD), multiple types of clinical imaging modalities have been combined with nanoparticle platforms^1,2^. These have improved ability to understand tumor microenvironment and quantify tissue drug concentrations^3–5^. However, current IGDD platforms suffer from interference by target tissue movement, scattering and image blurring for targets that are farther away and low resolution. These combine to limit clinicians’ ability to define undertreated regions with nanoparticles^6–11^. To address these IGDD barriers, in this study we developed a spectral Fiedler field (SFF)-based computational technology for enhancement of image sensitivity and spatial location of nanoparticles in solid tumors.

The proposed SFF methodology utilize graph and matrix theories to assess changes in surface topology from baseline. To do so, the deviations from reference geometry (i.e. subtle contrast changes vs. baseline) are transformed as quantifiable-flooded contour plots following nanoparticle injection. This innovative feature of SFF precisely measures the mismatch in tumor contrast in solid tumors over-time. We investigated this approach in murine colon cancer model utilizing ultrasound (US) imageable liposome as model nanoparticles. Ultrasound-imageable liposome are synthesized by encapsulation of phase-change contrast agents, consisting of nanodroplets of liquid perfluorocarbons (PFCs), emulsion^12^ (and other particles)^13^. The relatively small size of liposomes (100–300 nm) enables passive accumulation within tumors via the enhanced permeability and retention (EPR) effect^14^. However, the encapsulated PFC emulsions in liposomes is incompressible in a liquid state, and produce poor oscillation, backscatter, and image sensitivity in the ultrasound field^15–17^. Also, when stabilized by a lipid shell, the Laplace pressure, which is the pressure difference between the inside and the outside of an ultrasound (US) contrast agent (perfluoropentane, PFP) changes with the boiling temperature^18^. Thus, their poor resolution in the liquid state, and dynamic changes in the contrast with temperature can be an excellent model system for understanding the feasibility of SFF imaging approaches of nanoparticles in solid tumors. Our *in vivo* data suggest that the innovative SFF topological imaging approach has high sensitivity of detection for clinical applications.

## Method

### Algorithmic development of the topological tumor imaging platform

In order to quantify the changes in tumor topology over time, the first step of this proposed approach was to represent the original image ${\boldsymbol{X}}$ as a network graph *G*, i.e., achieve the mapping ${\boldsymbol{X}}\mapsto G$. Considering an image ${\boldsymbol{X}}$ with *M* by *N* pixels, which can also be represented as ${\boldsymbol{X}}=[{x}_{1},{x}_{2}\,\cdots \,{x}_{{\rm{N}}}]$, where each *x*_*i*_ is a column vector representing the pixel intensity of column *i*. Graph *G* was created by considering each column vector of ${\boldsymbol{X}}$ as one node of *G*, and the connectivity of *G* was determined by the distance between each pair of nodes. The distance between a pair of nodes *i* and *j* is noted as *ω*_*ij*_ was computed using a kernel function Ω. We utilized the radial basis kernel Ω^19^, and the constitutive equations as follows:

1 $${\omega }_{ij}={\rm{\Omega }}({x}_{i},{x}_{j})={e}^{-(\frac{k({x}_{i},{x}_{j})}{2{\sigma }^{2}})}\,\forall \,i,j\in \{1\,\cdots \,N\}$$

where ${k}({x}_{i},{x}_{j})={\Vert {x}_{i}-{x}_{j}\Vert }^{2}$ is the squared Euclidean distance between the two vectors; and *σ* is a free parameter in this study.

A threshold value *r* was applied to determine if two nodes are connected or not. Namely, two nodes whose distance was less than *r* were considered to be connected (see Equation (2.a)), i.e., there was an edge between these two nodes; otherwise, not connected, i.e., no edge. The connection relationship between each pair of nodes was summarized by a similarity matrix **S**, as shown in Equation (2.b).

2.a $${\rm{\Theta }}({w}_{ij})={s}_{ij}=\{\begin{array}{c}1,{w}_{ij}\le r\\ 0.{w}_{ij} > r\end{array}$$

2.b $${{\bf{S}}}^{N\times N}=[{s}_{ij}]$$

This allowed us to convert a solid tumor-image into an un-weighted undirected graph *G*, which can be represented by **S**.

Once the tumor image ${\boldsymbol{X}}$ was transformed as a graph *G*, the relevant topological information were extracted from *G*, which was subsequently used for quantifying the contrast of ${\boldsymbol{X}}$. To achieve this, the graph topological invariant Fiedler value (*λ*_2_)^20^ was used as a quantifier for ${\boldsymbol{X}}$, as follows.

3 $${d}_{i}=\sum _{j=1}^{N}{s}_{ij}\,\forall \,i,j\in \{1\,\cdots \,N\},$$

4 $${{\boldsymbol{D}}}^{N\times N}\mathop{=}\limits^{{\rm{def}}}\,{\rm{diag}}({d}_{1},\cdots ,{d}_{N}).$$

5 $${{\boldsymbol{ {\mathcal L} }}}^{N\times N}\mathop{=}\limits^{{\rm{def}}}\,{{\boldsymbol{D}}}^{-\frac{1}{2}}\times ({\boldsymbol{D}}-{\bf{S}})\times {{\boldsymbol{D}}}^{-\frac{1}{2}}\,{\rm{where}}\,{{\boldsymbol{D}}}^{-\frac{1}{2}}={\rm{diag}}(1/\sqrt{{d}_{1}},\cdots ,1/\sqrt{{d}_{N}}).$$

6 $${\boldsymbol{ {\mathcal L} }}v={\lambda }^{\ast }{\bf{v}}.$$

Equations (3) and (4) constructed the degree matrix ${\boldsymbol{D}}$, which was a diagonal matrix to represent the number of edges attached to each vertex. The Laplacian transformation based on Equations (5) and (6) was performed to compute Eigen spectrum of the Laplacian matrix ${\boldsymbol{ {\mathcal L} }}$, which was generated from degree matrix ${\boldsymbol{D}}$ and similarity matrix **S**. The smallest non-zero eigenvalue (λ_2_) was termed Fiedler value^21^. From graph theory perspective, *λ*_2_ is an effective measurement of graph connectivity. Except pathological scenarios that rarely occur in practical circumstances, the Fiedler value was bounded between 0 and 1, i.e., 0 < λ_2_ <1^20^.

### Synthesis of ultrasound imageable liposomes (abbreviated as E-LTSL)

Detailed scanning electron microscopy (SEM) and *in vitro* as well as *in vivo* characterization contrast of echogenic low temperature sensitive liposomes (E-LTSL) have been reported in the literature^22,23^. PFP (99%, Exfluor Research Corporation, TX, USA) was used as the ultrasound contrast agent in this study. Monostearoyl-2-hydroxy-sn-glycero-3-phosphocholine (MSPC), 1,2-dipalmitoylsn-glycero-3-phosphocholine (DPPC), and 1,2-distearoyl-sn-glycero-3-phosphoethanolamine-N-[methoxy (polyethylene glycol) 2000] (DSPE-Mpeg 2000) were obtained from Corden Pharma Corporation (CO, USA). For drug loading, doxorubicin was used as a model agent, and was obtained from LC laboratory (MA, USA). Briefly, lipids (composition: DPPC, MSPC, and DSPE-mPEG2000 in the molar ratio of 85.3:9.7:5.0) were prepared by hydration of a lipid film followed by the extrusion method. Lipid mixtures were dissolved in chloroform. The solvent was evaporated and the resulting lipid film was hydrated in citrate buffer (pH 4.0) mixed with 1,3-propanediol (1,3-PD (0.65 M, for PFP emulsification)) at 55 °C for 30 min and extruded five times through double stacked 200 nm polycarbonate filters to yield a final lipid concentration of 50 mg lipid/ml (80.8 mM)^24^. A PD-10 size-exclusion column equilibrated with 5–10 column volumes of 1x phosphate buffered saline (PBS) was used to remove free 1,3-PD from the outside of the liposomes. Encapsulation of Doxorubicin into the LTSLs was carried out using the pH-gradient loading protocol described by Mayer *et al*.^25^. PFP was loaded into the LTSL to synthesize E-LTSL using a one-step sonoporation method. Briefly, 2 mL of the liposomal formulations were incubated under continuous sonication (~20 kHz) in 3 mL vials along with PFP (boiling point 30 °C; 20 μl/100 mg lipid) for 1–2 min. PFP and LTSLs were kept cold prior to being combined, and the sonication bath was kept at 10–15 °C to minimize PFP vaporization. Free PFP was removed using a PD-10 column. This method was repeated at least in triplicate (n = 3) for evaluation. E-LTSL were characterized for size (z-average), polydispersity index and zeta potential using dynamic light scattering (DLS) with a 90 plus PALS Nanobrook device (Brookhaven Instruments, Holtsville, NY, USA). Briefly, 10–20 µl of E-LTSL was added to 2 ml of PBS in a cuvette, and DLS measurements were recorded at room temperature. An average of five measurements was taken, and the mean size and standard deviation were calculated.

In this study, we used Doxorubicin-encapsulated temperature sensitive liposomes that are sensitive to mild, non-destructive temperature elevations above normal body temperature. Additionally, to provide imaging and precision-warming capabilities, we co-encapsulated LTSLs with PFP, an echogenic contrast agent. This can permit *in vivo* tracking of liposome distribution using an ultrasound device and improve/fine-tune real-time control of drug delivery. PFP transitions from a liquid (~29 °C) to an echogenic state at body temperature, which can allow us to track LTSL redistribution during locally-applied hyperthermia to induce Dox release from the liposomes. Also as mentioned in Sec. 1, when stabilized by a lipid shell, the Laplace pressure substantially increases its boiling temperature. This is caused by the surface tension at the interface between PFP and bulk liquids. This predictable property of PFP boiling point changes can be hypothetically applied for nanothermometry and nanomonitoring of drug delivery.

### *In vivo* imaging in mouse colon cancer

All animal-related procedures were approved and carried out under the guidelines of the Oklahoma State University Animal Care and Use Committee. C26 cells were kindly provided by the National Cancer Institute, and established in athymic nude mice as previously described^23,26^. Briefly, confluent C26 cells grown as a monolayer in RPMI medium supplemented with 10% v/v fetal bovine serum and 1% v/v streptomycin/penicillin were harvested, washed, and diluted with sterile cold PBS at 0.5 × 10^5^ cells/50 μl. Next, 50 μl of cell inoculum was injected subcutaneously in the thigh region of the mouse hind leg with a 25-gauge needle (BD; Franklin Lakes, NJ, USA). Mice were monitored and tumor growth was measured by serial caliper measurements (General Tools Fraction+™, NY, USA). Tumor volumes were calculated using the formula (length × width^2^)/2, where length is largest dimension and width is the smallest dimension perpendicular to the length. Tumor imaging was initiated at a volume of 300–400 mm^3^. For imaging, mice were anesthetized with 2–5% isoflurane and then secured on a heating stage maintained at 37 °C (Stryker, MI, USA). All ultrasound imaging was conducted using a VisualSonics Vevo 2100 ultrasound MS550D transducer (22–55 MHz, Fujifilm, Toronto, ON). For imaging, the transducer was placed in a stationary position oblique to the tumor with special clamps built in-house. Mice were injected with 100 μl of E-LTSLs (~10 mg total lipid) followed by 50 μl of saline flushed through a catheter placed in the tail vein. Tumor imaging was conducted at 37, 39 and 42 °C in separate cohorts (*n* = 5–6) of mice by isolating only the tumor-bearing leg in specialized holders to understand topological features as a function of temperature.

### Motion compensation for SFF-methodology

To assess motion correction in the *in vivo* model and determine the variation of image intensity over time, a cine acquisition of 100 frames at 20 f/sec was acquired at different time points up to 15 min in our mouse model. A region of interest (ROI) encompassing the tumor was defined in the first frame and tracked in subsequent images by applying a rigid translation and rotation to the ROI to maximize the similarity between ROIs in each successive frame pair. Multiple ROIs were selected to include the region of the tumor as well as other areas with high feature variations. In cases the tumor region did not have sufficient features to reliably compute similarity measures, alternative feature-rich ROIs were used. The rigid transformation computed using the alternative ROI was then applied to the tumor ROI. The similarity between ROIs was computed using Normalized Cross-Correlation (NCC). Once motion corrected, ROI sequences were analyzed over time as median intensity over the ROI normalized to the range [0, 1]. This averaging method provided implicit regularization for noise and allowed determination of general characteristics of the ROI (peak intensity, rise time).

### SFF imaging of tumor liposome contrast

**Figure 1:**
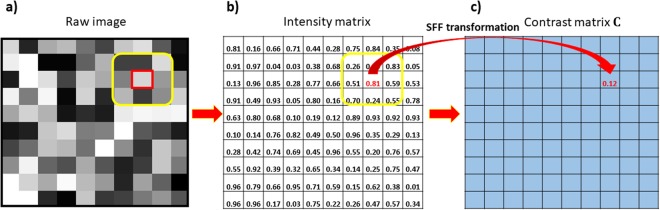
Procedure of the SFF transformation. (**a**) A raw image in gray scale; (**b**) matrix representation of the raw image; (**c**) contrast matrix generated using SFF. For a given gray scale image with its matrix form, SFF operation is performed on a pre-defined small region centered at every single pixel, and the contrast matrix ***C*** is generated accordingly. Contrast matrix ***C*** is finally processed using a low pass filter to remove the noise, and the resulting matrix is termed SFF map ***F***.

In order to achieve liposomal mapping in colon tumor over time, we designed a graph quantification framework based on Fiedler value *λ*_2_, wherein a pixel-based transformation of images termed Spectral Fiedler Field (SFF) was performed (Fig. 1).

**Figure 2:**
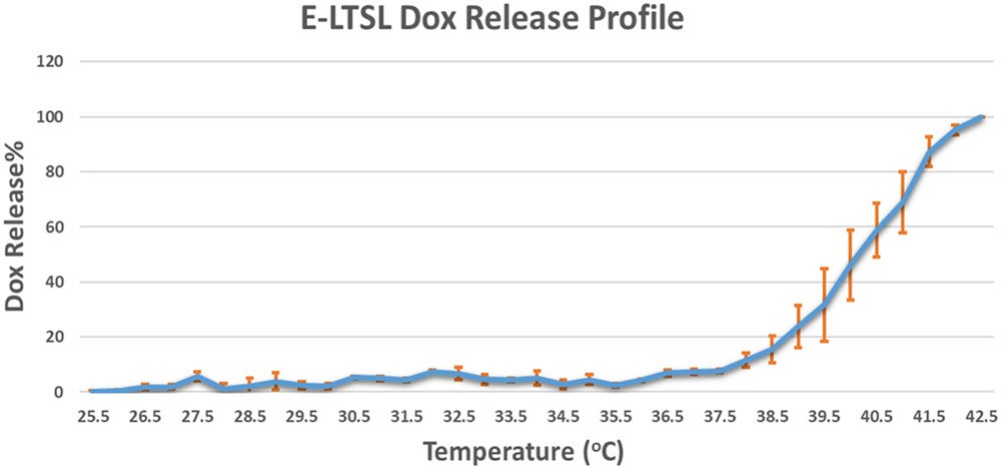
Dox release from E-LTSL as a function of temperature showed >95% release at >40 °C.

The implementation of SFF for an ultrasound image (Fig. 1a) includes four steps: (1) Define neighborhood region ${{\mathscr{X}}}_{ij}$ of each pixel *p*_*ij*_ (highlighted in Fig. 1b); (2) Based on region ${{\mathscr{X}}}_{ij}$ to construct a graph *G*_*ij*_ using the proposed SFF operation presented in Sec. 2.1; (3) Quantify the contrast of pixels within region ${{\mathscr{X}}}_{ij}$ by using the Fiedler value (i.e., *λ*_2_) of *G*_*ij*_, denoted as *c*_*ij*_ (exemplified as “0.12” in Fig. 1c) corresponding to *p*_*ij*_, and a contrast matrix **C** can be constructed by {*c*_*ij*_}; (4) Apply low-pass filtering^27^ to **C**, and the resulting SFF map is termed as **F**.

Low-pass filtering was applied to reduce noise within an image, also known as image smoothing. Since the contrast map is usually noisy, using a low pass filter retained the low frequency information (i.e., nanoparticle concentration) while reducing the high frequency information (i.e., noise). Next, by using the SFF transformation for the ultrasonic images, for a given time *t* after drug injection, the contrast variation contour maps **V**_*t*_ was estimated by

7 $${{\bf{V}}}_{t}={{\bf{F}}}_{t}-{{\bf{F}}}_{0}$$

where **F**_0_ is the SFF map of the B-mode image before injection. This proposed real-time contrast mapping technique named as “spectral Fiedler field-ultrasound (SFF-US) imaging”, allowed determination of the contrast/change of SFF in tumor in real-time, and also generated on-the-fly contour maps of liposomal distribution over time “on-demand”. Additionally, to determine if SFF-US imaging correlates with tumor vascular regions, E-LTSL was injected in mice bearing colon tumors (5 mg/kg Dox). Following E-LTSL injection and 20 min hyperthermia, a region of tumor matching the footprint of ultrasound transducer image from harvested tumors were sectioned. Three (7-µm) serial sections were mounted per slide, and the endothelial cell marker cluster of differentiation 31 (CD31) can be used to determine blood vessel density as published earlier^28^. To estimate Dox penetration, 2D isotropic diffusion model was applied using the parameters and using SFF intensities as a relative scale. The vessel centers were computed using SFF-US mapping **V**_*t*_ by applying a consistent threshold as well as morphological operators. The centroid of the resultant isolated patch was used as vessel center. The tumor boundaries were masked by manually defining a boundary mask in ultrasound B-mode images, and Dox distribution was determined. Image acquisition and display parameters were constant for different treatments to allow for qualitative comparison. Whole-section digital histological scans were acquired via a 4X objective on an Olympus ZDC2 IX81 fluorescence microscope equipped with motorized scanning stage, and mosaic stitching metamorph software.

### Quantification of liposome contrast changes in tumor

The spatial distribution of the injected micro or nano-bubble is typically not uniform, and the overall contrast measurement of the whole tumor cannot represent the exact variations in liposome contrast locally. Therefore, as opposed to a single quantifier in Sec. 2.1, a spatial contrast mapping for ultrasound image was utilized to effectively describe the distribution contrast variation of ultrasound images. Since the ultrasound images contained information about variation of pixel of the image, the Fielder value (*λ*_2_) was able to capture subtle aspects in the image pixel variations that were not discernable from the existing method^13^. More pertinently, it was inferred that higher the pixel variation of the region, the larger the Fiedler value (*λ*_2_)^29,30^. Hence, a comparatively large Fielder value signified a region with high contrast. This was then applied to quantify the contrast changes of the ultrasound images. Thus, based on the SFF mapping ***F***, a quantified ultrasound image contrast *C* was measured as given below

8 $$C=\frac{{\sum }_{(i,j)\in R}{c}_{ij}}{{\sum }_{(i,j)}{I}_{(i,j)\in R}}$$

where *R* indicates the ROI of the ultrasound image and *I* is an indicator function (*I* = 1 if pixel (*i*, *j*) belongs to ROI, and 0 otherwise); *c*_*ij*_ is an element of the contrast matrix **C** of the ROI (Fig. 1). Therefore, the definition of image contrast *C* can be interpreted as the SFF intensity within the ROI divided by the area of ROI.

## Results

### Characterization of E-LTSL

The hydrodynamic diameter of E-LTSLs at room temperature (25 °C) was 165 ± 1.0 nm, and the polydispersity index values were 0.13 ± 0.01, respectively. At optimized PFP concentrations, E-LTSLs encapsulated 70% of added Dox. Percent Dox release from E-LTSL was minimal (<5%) at 25–39 °C (Fig. 2); was followed by a more gradual release at 40 °C (~20%), and was rapid and complete (>95%) near the temperature giving maximum release rate (~41–42 °C).

### Generation of on-the-fly maps of liposomal SFF contrast/accumulation in tumors

**Figure 3:**
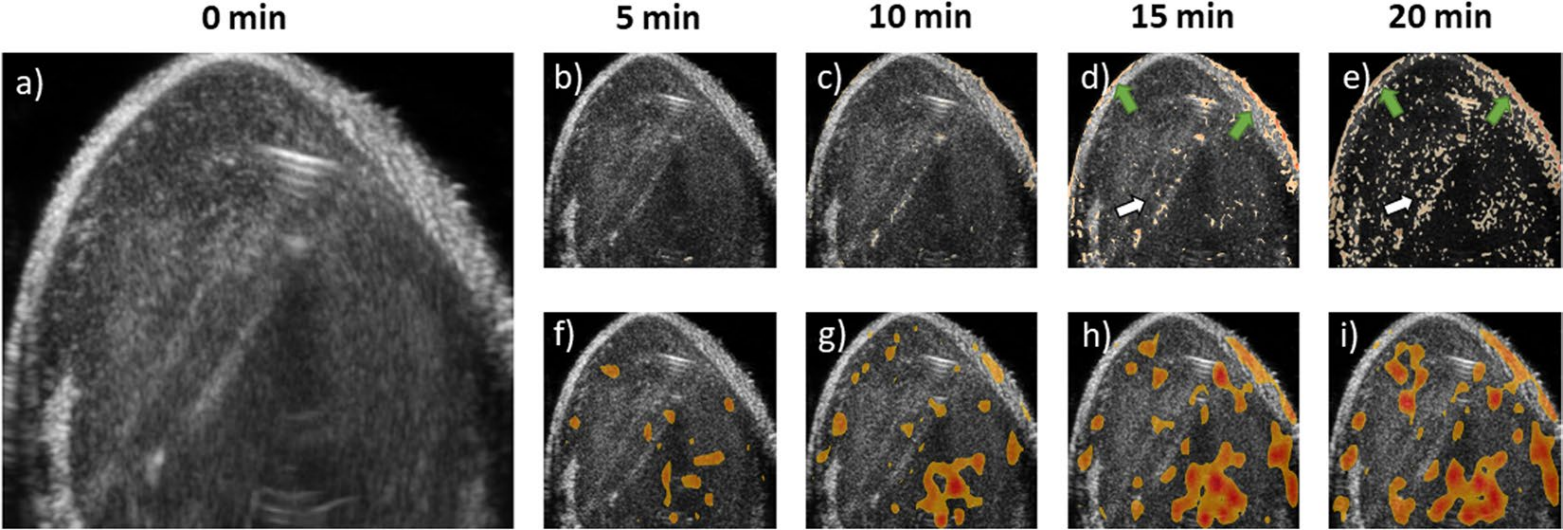
(**a**) Liposomal contrast in tumors at time 0; (**b**–**e**) Spatial ultrasound mapping after injection (5–20 min) based on conventional methods, with pixel by pixel subtraction for the motion compensated ultrasound images showed high noise; (**f**–**i**) On-the-fly maps of liposomal contrast/accumulation generated by SFF-US imaging technique (5–20 min) resist the effects of noise to pinpoint liposome accumulation.

Compared to the conventional direct pixel by pixel intensity subtraction method^13^ that gave noisy result, the SFF-US mapping readily generated on-the-fly maps. In conventional method, minute variations in imaging intensity may not be directly related to change in state or concentration of nanoparticles. For instance, sources such as imperfect motion compensation, imaging artifacts during acquisition or simply noise in the ultrasound image can get aggregated in the liposome contrast quantification in tumor. A typical example of such artifacts and noise (white arrows) is highlighted in Fig. 3b–e over a 20 min imaging period. These are complicated further by imperfect matching of the skin surface during the motion compensation (green arrows). Interestingly, both of these spurious contrast signal are removed by the SFF-US imaging method (Fig. 3f–i).

### Correlation of SFF-US imaging with vascular and drug contrast

**Figure 4:**
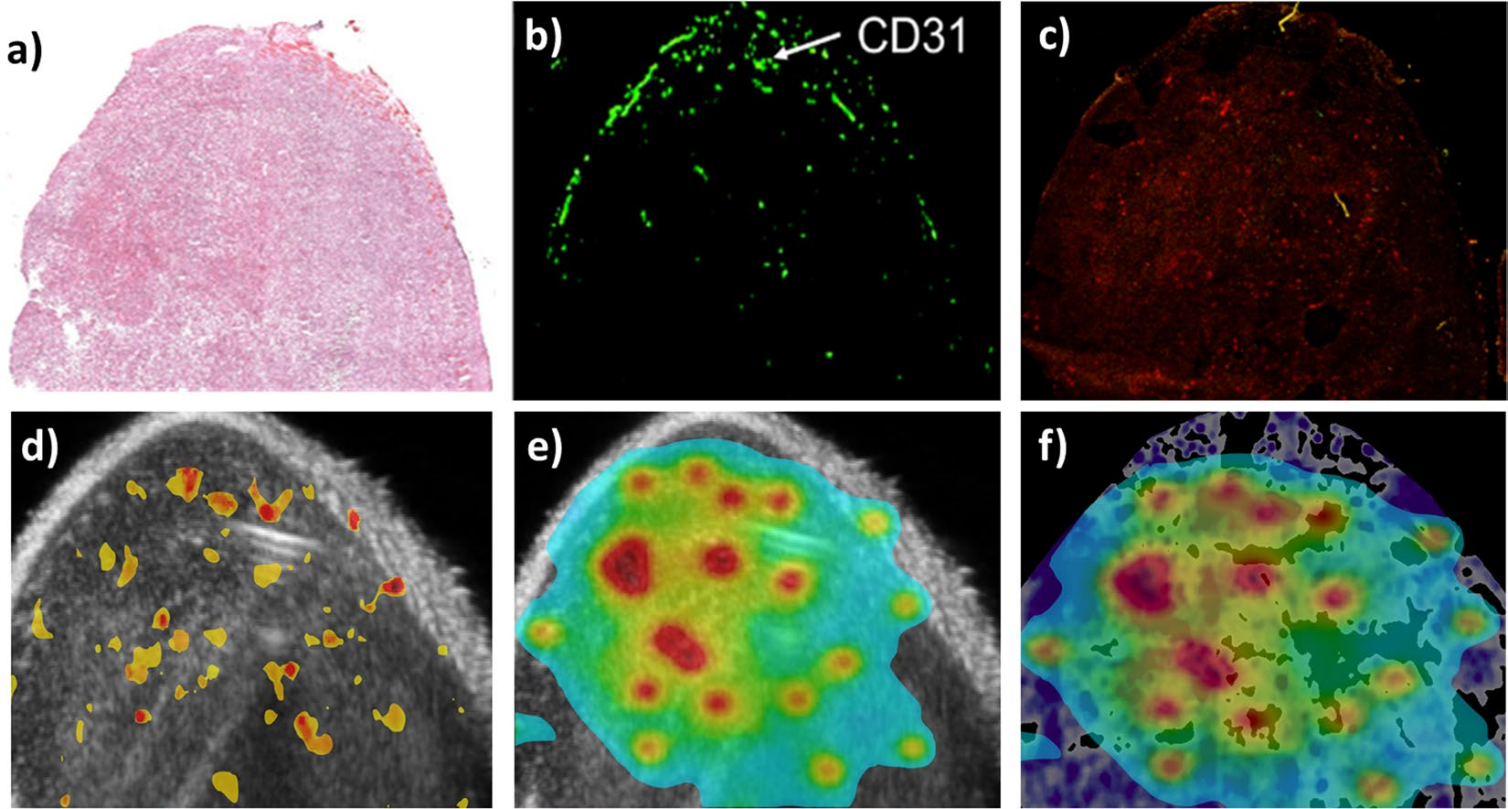
(**a**) Hematoxylin and eosin (H&E) image of colon tumor; (**b**) CD31 endothelial cell marker; (**c**) Dox fluorescence in tumors; (**d**) SFF imaging of echogenic liposomes enhanced ultrasound contrast in tumor regions that were positive for CD31 endothelial cell marker; (**e**) Estimated relative drug diffusion using the positive regions in SFF imaging. Values for estimated Dox distribution normalized between 0–1 range, where blue represents 0 and red represents 1; (**f**) Normalized estimated Dox distribution overlaid on Dox fluorescence image that has been aligned to the ultrasound image using skin surface as fiducial. Dox fluorescence is show in sequential shades of purples, where as Dox distribution is shown in spectral shades of blue-red. Dox fluorescence has been filtered with a median filter to reduce noise in the image.

Hematoxylin and eosin (H&E) staining suggested minimal necrosis in the liposome treated tumors (Fig. 4a). Tumor microvessel density determined by immunofluorescence staining against CD31 demonstrated angiogenesis (Fig. 4b). The correlation of CD31 and liposome localization using SFF was excellent (Fig. 4c). Because Dox permeate from vessel into the tumor interstitial, we also determined the doxorubicin fluorescence in the region of interest. Dox signal (red) in the liposome treated tumor region extended beyond the blood vessels and the fluorescence intensity matched excellent with the predicted SFF drug distribution model (Fig. 4d–f).

### Quantification of ultrasound contrast in colon tumor by SFF

**Figure 5:**
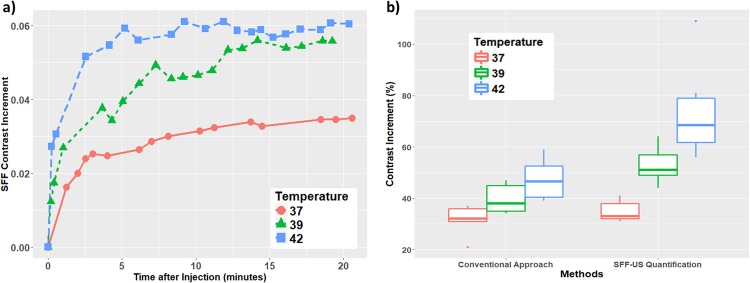
(**a**) A temperature dependent contrast increment (divided by the area of ROI) based on SFF-US quantification for colon tumor noted with time under the temperatures of 37 °C (low temperature), 39 °C (medium temperature) and 42 °C (high temperature), respectively. The signal under 42 °C is much more significant than that under 37 °C, thereby suggesting a correlation of Laplace pressure mediated increase in tumor intensity with temperature; (**b**) Comparison between SFF-US quantification and conventional approach based on the percentage of tumor vascular contrast increment (%) in terms of the defined SFF-US quantification under 37 °C, 39 °C and 42 °C, respectively. Based on the experimental results, the SFF-US quantification provided a significantly enhanced contrast signal than the conventional method and it also showed a much better performance to distinguish the increment difference under different temperature for tumor mapping purposes.

To quantify the performance of the proposed SFF-US imaging technique, we measured the SFF-US contrast (i.e., the value of C introduced in Sec. 2.6) of mouse colon tumors injected with E-LTSL at 37 °C, 39 °C and 42 °C, respectively for 20 min. As shown in Fig. 5a, for a single animal experiment, the higher temperature caused larger increment of the ultrasound tumor contrast due to higher variations in Laplace pressure, as indicated by the increment of SFF-US contrast based on ROI area (the SFF-US contrast increment within the ROI divided by the number of pixels of the ROI). After multiple animal experiments, the comparison illustrated that the SFF-US offered a much stronger signal than the conventional method under the same condition (Fig. 5b), suggesting that the SFF method is relatively more sensitive and specific compared to currently available methods.

To quantify the comparison results, two-sample *t*-test was applied to determine the existence of the difference between each pair of two temperature treatments^31^. The percentage of image contrast increment defined both conventional method and SFF-US. The criterion of two-sample *t*-test is based on the p-value that indicates the significance of difference. For the SFF-US quantification, the testing results showed that all the p-values are lower than *α* (0.05) when comparing the contrast increment under 37 °C, 39 °C and 42 °C, which are consistence with Fig. 5b. On the contrary, the capability of conventional method to distinguish the increment difference is much lower than the proposed SFF-US in terms of the p-value (Table 1).

**Table 1:** The results of two-sample t-test comparing the difference of contrast increment percentage under different temperatures.Under *α* = 0.05, the conventional method can only identify the difference between 37 °C and 42 °C, but SFF-US quantification is able to detect the signal difference among all of the three temperature conditions.

	37 °C VS 39 °C	39 °C VS 42 °C	37 °C VS 42 °C
Conventional Method	0.062	0.11	0.0054*
SFF-US Quantification	0.0035*	0.046*	0.0036*

The number of experimental animals in each condition is 5 with 37 °C, 5 with 39 °C and 6 with 42 °C.

## Discussion

The objective of this study was to test the feasibility of SFF-US methodology for tumor mapping of nanoparticle in a murine colon cancer model. The developed SFF mapping approach is based on network connectivity *λ*_2_ quantification techniques (i.e., Fiedler value based). *λ*_2_ has been applied previously for quantification of surface roughness, since the image captured from surfaces with higher roughness correlated with higher connectivity, i.e., larger *λ*_2_. The testing performance showed that the measurement accuracy of this method was in line with the industry requirement. *λ*_2_ was also fast to perform (within 0.1 s), by selecting images with a relative high frequency (at least 1 Hz) quickly, thereby significantly reducing the computational cost. As this approach is non-invasive, large algorithmic computations utilizing 3D point cloud are achievable without dramatically influencing the contrast imaging^32^. This is achieved by eigenvalues of the Laplacian matrix (not only *λ*_2_) as extracted features from 3D point cloud data followed by a supervised machine learning algorithm for classification of the severity of dimensional variation. Thus, SFF is an innovative technique to ascertain quality of images quickly instead of scanning an entire sample.

The key innovation of this paper lies in combining feature extraction with spatial contrast mapping (a single value) for tumor contrast mapping applications. Our data suggest that SFF is robust and sensitive to various temperature (37 and 42 °C) with the model ultrasound imageable liposome nanoparticles. It may be noted that echogenic liposomes, nanodroplets, and solid nanoparticles (100–300 nm) migrate more easily through the tumor blood vessel into the interstitium by enhanced permeation and retention effect (EPR) mediated drug delivery in various tumors^33–35^. However, the high-resolution ultrasound detectability of nano-sized contrast agent at reduced size is challenging^14^, and coalescence into microbubble size, or conversion of contrast agent into gaseous bubbles by changing the Laplace pressure inside nanoparticles are needed in most cases^36,37^. Our results show that SFF-US imaging reduces the bright and noisy signals (from skin) during ultrasound imaging to provide a consistent measurement (with very small variation) via robust network connectivity quantification (Fig. 5). Additionally, our motion compensation methodology and combination with spectral graph theory based B-mode image global quantification convert the motion compensated B-mode ultrasound images into a local contrast contour map via SFF image transformation to pinpoint/quantify the local area with the most contrast variation, directly related to the presence of liposome/drugs in tumors (Fig. 4).

In summary, this work developed a new SFF-US contrast imaging platform based on graph theory for mapping of nanoparticle in the tumor. SFF-US imaging was able to effectively pinpoint the areas that have the most liposomal contrast variation in colon cancer. Compared with the existing method, the developed SFF-US imaging method provided a more robust and sensitive measure for the identification of contrast variation. This technology can potentially improve IGDD contrast monitoring in solid tumors.

Our paper has some limitations. First, we did not investigate the feasibility of SFF in tumors of various architecture (fibrotic vs. vascular; immunocompetent syngeneic vs. immunocompromised athymic model, heat vs. no heat, etc.). Studies are currently underway to understand real-time monitoring of drug/liposome transport in variable tumor microenvironments. We also believe that the correlation of SFF imaging with other classes of drug can provide an innovative technology to understand the transport of conventional anticancer agents following intravenous therapy. Finally, mismatch of SFF/CD31 co-localization, and correlation of contrast imaging with other imaging modalities (MRI, PET) will inform on mechanistic differences in SFF functionalities in different clinical situations.

### Disclaimer

This feature is based on research, and is not commercially available. Due to regulatory reasons its future availability cannot be guaranteed.
